# Vehicular traffic effects on elk and white-tailed deer behavior near wildlife underpasses

**DOI:** 10.1371/journal.pone.0269587

**Published:** 2022-11-07

**Authors:** Mehdi Nojoumi, Anthony P. Clevenger, Daniel T. Blumstein, Eric S. Abelson

**Affiliations:** 1 Department of Ecology and Evolutionary Biology, University of California, Los Angeles, Los Angeles, CA, United States of America; 2 Western Transportation Institute, Montana State University, Bozeman, MT, United States of America; 3 La Kretz Center for California Conservation Science, Institute of the Environment and Sustainability, University of California, Los Angeles, CA, United States of America; Sichuan University, CHINA

## Abstract

Roads fragment animal populations, vehicles kill and injure animals, and traffic may affect animal behavior. Mitigation efforts (e.g., wildlife underpasses) are constructed to prevent fragmentation and reduce wildlife-vehicle collisions. However, little is known about traffic’s proximal effects on wildlife behavior and use of mitigation measures. We quantified the time that elk (*Cervus elaphus*) and white-tailed deer (*Odocoileus virginianus*) allocated to foraging, vigilance, and flight behavior before and after vehicle passage. Both species increased vigilance and flight behaviors and reduced time spent foraging in response to vehicles. Both species were more likely to move through the underpass if they exhibited foraging behavior; we also found a marginally significant trend that animals were less likely to use the underpass after vigilance behavior. Knowledge that vehicle movement influences wildlife behavior underscores the importance of consideration given to road and crossing structure design. Additionally, findings of species-specific response to vehicle passage are important in understanding potential fitness consequences of anthropogenic disturbance.

## Introduction

Roads and highways threaten species population viability as well as overall biodiversity by destroying habitat and fragmenting populations [1]. Wildlife-vehicle collisions have increasingly been an issue of concern for many state and provincial transportation agencies [2, 3]. Mitigation efforts, such as the construction of underpasses and overpasses, are measures to reduce some of the negative effects of roads [4–6].

The efficacy of wildlife-crossing structures depends on both physical characteristics as well as the auditory and visual stimuli in the surrounding environment [7, 8]. Auditory and visual stimuli created by passing vehicles, unlike physical dimensions of underpasses and overpasses, are a transient feature [9]. Understanding behavioral responses of animals to these stimuli near wildlife crossing structures is essential to determine their efficacy. For instance, traffic-associated auditory and visual stimuli can repel wildlife from areas that are intended to serve as points of connectivity [7, 10] and human-generated noise is known broadly to have a host of deleterious effects on wildlife [11]. As a result, it is possible that crossing structures alone, especially along relatively impermeable roads, may be underutilized without additional infrastructure to block sounds or sights of passing vehicles because of animal response to traffic-related stimuli.

Several studies have evaluated the effectiveness of physical characteristics of wildlife structures [5, 12, 13]. For instance, Clevenger and Waltho (2000) previously documented the effects of width, length, and other physical dimensions of underpasses on carnivores and ungulates. Barrueto et al. (2014) found that wildlife can habituate to some types of disturbances (e.g., vehicle traffic), but remain sensitive to others (e.g., foot traffic at wildlife crossing structures), and that crossing structure designs in Banff National Park were capable of buffering some of the potential aversive stimulus produced by roads (e.g., light, noise). Some studies have examined wildlife behavior during crossings [14, 15]. Although previous research finds relationships between traffic volume and deer or elk use of passage structures, for example see [10, 16, 17], we are aware of no previous study that examined the immediate vehicular effects on behavioral responses of animals near and/or using wildlife underpasses.

We examined the effects of traffic volume (number of vehicles passing per 15 second window), vehicle type (passenger vehicles vs. large vehicles including semi-trucks, buses, recreational vehicles) on the immediate behavioral responses of elk (*Cervus elaphus* Linnaeus, 1758) and white-tailed deer (*Odocoileus virginianus* Zimmermann, 1780) using two wildlife underpasses in Banff National Park, Alberta, Canada. We measured the time ungulates allocated to different behaviors before and after vehicles passage. We focused on the following behaviors: foraging, vigilance, and fleeing (i.e., running). We asked whether species identity, and the number and type of passing vehicles modified these behaviors and whether there was a relationship between the change in time allocated to each behavior after vehicle passage and the probability of crossing.

## Materials and methods

### Study area

**Fig 1:**
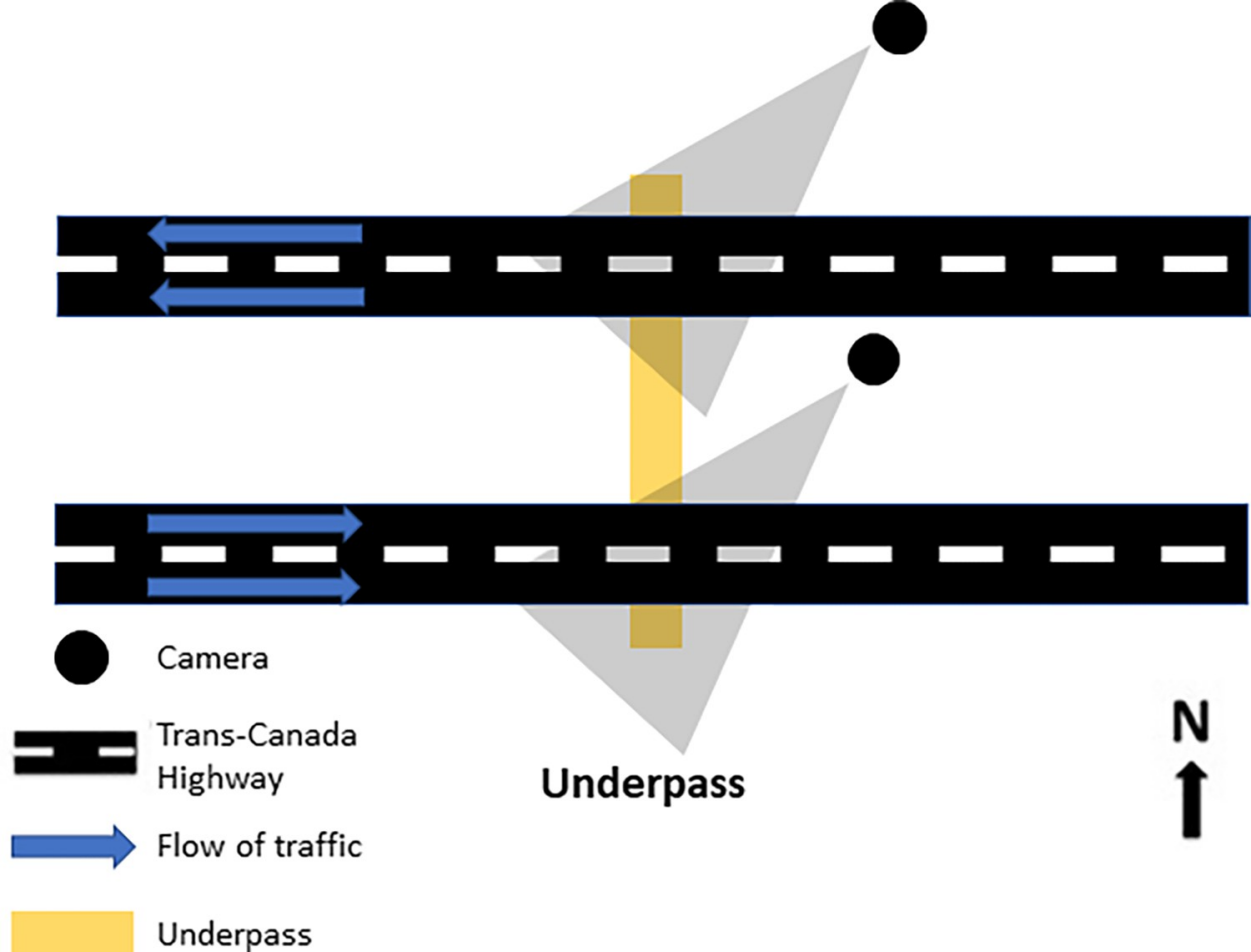
Location and orientation of cameras to film deer and elk at wildlife underpass in Banff National Park, Alberta. Flow of traffic in the top two lanes are westbound while lower two lanes depicted are eastbound.

Videos were recorded along the Trans-Canada Highway in Banff National Park (BNP), Alberta, Canada, located approximately 120 km west of Calgary. The Trans-Canada Highway runs within the Bow River Valley, which is located in the continental ranges of the central Canadian Rocky Mountains [4]. It is a major transportation corridor through the national park, carrying an annual average of 17,000 vehicles per day (Parks Canada 2008). The studied underpass is located in the first 45 km of the highway from the eastern park boundary where four lanes of traffic (two eastbound and two westbound lanes) are bordered on both sides by a 2.4 m high wildlife exclusion fence (Ford et al. 2010; Fig 1). The underpass is located on a divided section of highway, thus consisting of two tunnels, one under each direction of traffic. The 3.5 m tall, 9 m wide, and 40 m long underpass had been in place for nearly twenty years (at the time of the study, providing ample time for animals to learn to use it). Elk and white-tail deer population are healthy in the Banff National park with elk densities ranging between 1 and 10 elk/km^2^ [18]. Elk are partially migratory while white-tail deer are not migratory.

### Recording behavior

We set up two video cameras (Sony Super 8; Sony Corporation of America, New York, NY, USA) that were checked daily and motion-activated using TrailMaster active infra-red sensors (Goodson & Associates, Inc. Lenexa, KS) [19]. Cameras were positioned 1 m off the ground and 20–25 m from the underpass. One camera was set up on the north side of the wildlife underpass and recorded the underpass and westbound traffic (Fig 1). The other camera was set up beneath the median and recorded eastbound traffic. The video camera field-of-view included both area under the crossing structure and the road so that wildlife and vehicles were both captured. We used the camera on the north side of the underpass to record behavior and underpass use of elk and white-tailed deer (only the north side camera was used to assess behavior as it recorded behavior occurring when the animal was not actively using the underpass); we used both the north camera and the camera between the lanes of traffic to identify successful underpass crossings. The cameras recorded either the eastbound traffic or the westbound traffic; recorded vehicle passage was used to demarcate the time period before and after vehicle passage. Recordings took place from May to October 2004 and ran 24 hours per day and 7 days per week. A research permit for this work was obtained from Parks Canada as part of a larger multi-year study of road impacts on wildlife and efficacy of Trans-Canada Hwy mitigation measures. All sampling procedures (i.e., methods using remotely activated camera systems) were reviewed as part of obtaining a research permit. Remotely activated cameras were used as part of a non-invasive study design; therefore, no permits concerning animal welfare/ethics were required.

To record behavior, we used only video footage of animals walking towards the underpass, rather than those who emerged from the underpass in the middle and were making decisions to cross the second underpass structure on a divided section of the highway. To record passage rates, we focused exclusively on animals that began to move through the underpass and then either emerged from the other side (successful crossing) or fled in the direction they entered the underpass (unsuccessful crossing).

In some cases, more than one individual of a species was present. In these cases, we selected the individual who was initially closer to the camera (to minimize the amount the view of the animal would be obstructed). We excluded one observation where two elk and one deer crossed together. In some cases, more than one type of vehicle was present. In these cases, we selected the largest vehicle because we assumed that it produced the greatest stimulus intensity.

We quantified the amount of time spent in the following behaviors: foraging, vigilance (i.e., standing and looking), and flight (i.e., running). Foraging was defined as standing quadrupedally while animal’s head was down at the vegetation level. Looking was defined as standing quadrupedally while the head was fixed and not at vegetation level (i.e., foraging). Any change in the direction of the head was counted as a separate bout of looking. Running was defined as any speed above steady, slow locomotion. We also recorded whether animals used (i.e., crossed) the underpass.

We used JWatcher version 1.0 [20] to quantify the amount of time animals allocated to each behavior 8–15 s before, and an equal amount of time after, the first vehicle passed (i.e. 8–15 s) for videos containing both vehicles and deer or elk (S1). The number of seconds used varied (but was always between 8–15 s) as a function of when the camera first triggered and started recording; we used the longest duration available before a vehicle passed. We excluded videos that were separated by less than 15 min to avoid pseudoreplication.

### Statistical analysis

For our analyses we calculated the change in the proportion of time spent in a given behavior before and after the passage of vehicles. This ranges from 1 to -1 where positive values indicate increases in the behavior of interest after vehicle passage while negative values indicate a reduction of time spent in the behavior of interest after vehicle passage. Specifically, we calculated the difference in the proportion of time allocated to each behavior (e.g., foraging) using the following equation: (foraging behavior time (s) / total time after vehicle passage in the recording (s))–(foraging behavior time (s) / total time before vehicle passage in the recording (s)). For any given video, values were calculated for a given behavior only if the animal engaged in that behavior either before, after, or both before and after vehicle passage.

To further illustrate the interpretation of these values: 0 values indicate that the animal spent an equal amount of time engaged in the behavior of interest both before and after the passage of vehicles. A value of 1 indicates that an animal spent no time engaged in the behavior of interest before the passage of a vehicle but spent 100% of time after vehicle passage in that behavior. Conversely, -1 indicates that the animal spent 100% of time engaged in a behavior before vehicle passage that the animal spent 0% of time after vehicle passage. A plausible interpretation of this statistic is that vehicle passage may be resulting in an increase (positive values) or decrease (negative values) in behaviors where larger absolute values indicate the strength of the effect.

Three types of models were employed: one-way *t*-tests, linear models, and logistic regression models. All models were conducted using program R statistical software [21]. We conducted one sample t-tests with the null being that the mean of the change in proportion of time spent engaged in foraging, vigilance, and flight behaviors (before and after vehicle passage) is equal to 0. Linear models were fitted with the change in proportion of time spent in a behavior of interest (i.e., foraging, vigilance, flight) as the dependent variable. In all linear models, number of vehicles, type of vehicle, and species (deer or elk) were used as independent variables. Vehicle type fell into two categories: passenger vehicle and trucks (including other large vehicles such as busses and recreational vehicles). Each model was tested to ensure that it passed model assumptions for normality, independence of residuals (autocorrelation), and heteroscedasticity using the performance package [22]. Finally, logistic (binomial) regressions were fit with passage (equal to 1 if the animal used the crossing structure and 0 if the animal did not use the crossing structure) as the dependent variable. Species (deer or elk) and change in proportion of time spent in the behavior of interest before and after vehicle passage were used as independent variables. Again, model assumptions were tested using the performance package.

## Results

From a dataset containing 597 wildlife videos, 36 observations met our analysis criteria. Our final data set included observations of 21 deer and 15 elk crossings.

### Does mean time spent foraging, vigilant, or fleeing increase/decrease after vehicle passage?

**Fig 2:**
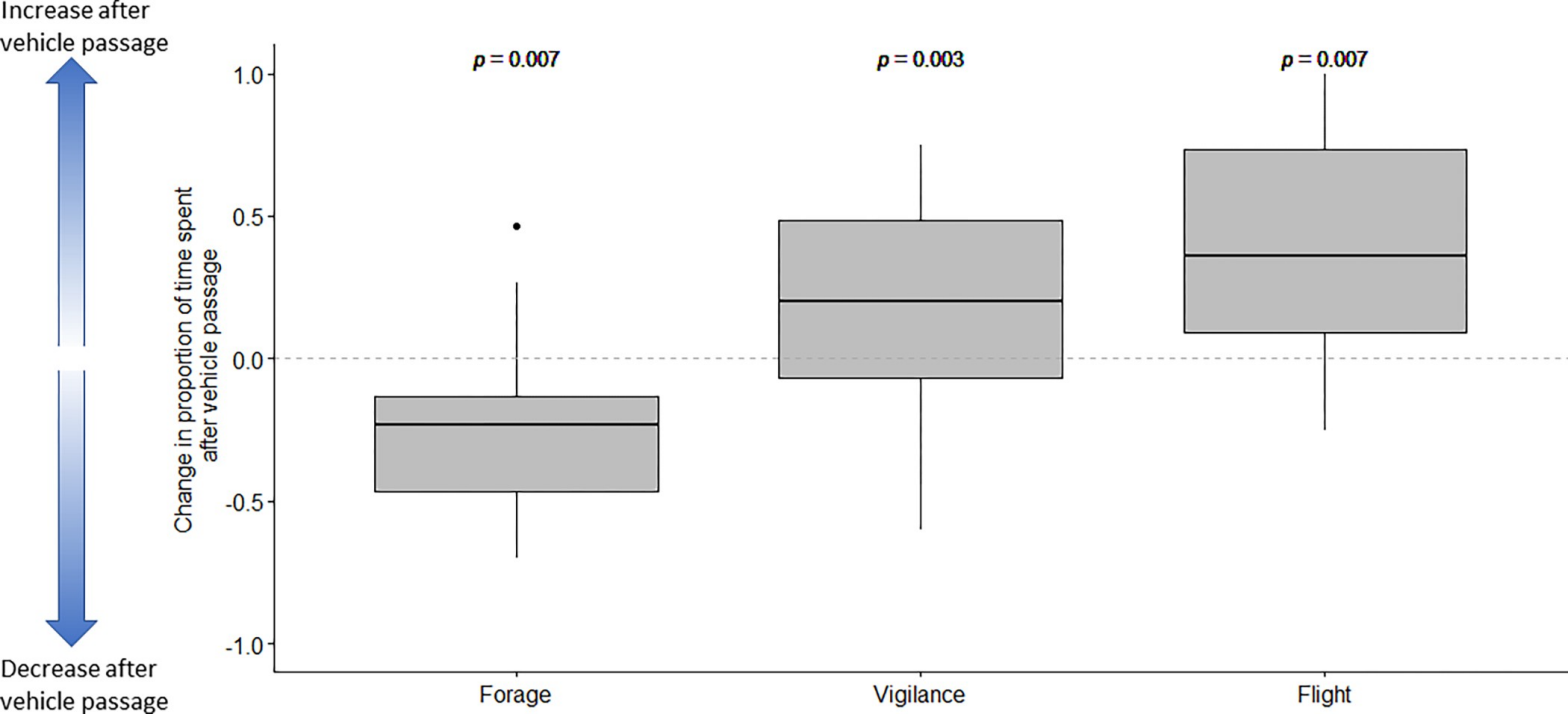
Change in proportion of time spent foraging, vigilant, and fleeing after vehicle passage. Larger positive values indicate an increase in proportion of time spent in any given behavior after vehicle passage (i.e., vigilance and flight behaviors) while smaller values (i.e., negative values) indicate a reduction in the proportion of time spent in a given behavior (i.e., foraging). Significant p-values (from a one sample t-test) indicate a that the sample mean is not equal to 0. Each boxplot visualizes the median, hinges representing the 25^th^ and 75^th^ percentiles, and whiskers (i.e., the largest value that is no further than 1.5 * IQR from the hinge).

We conducted one sample t-tests and found that, after vehicle passage, the mean change in the proportion of time spent foraging (M = -0.24, SD = 0.33) was significantly lower than 0 (where 0 indicates that there was no change in behavior after vehicle passage); this indicates a significant decrease in foraging behavior after vehicle passage; t = -3.08, p = 0.007 (Fig 2). Conversely, we found that, after vehicle passage, there was a significant increase in the mean change of the proportion of time spent being vigilant (M = 0.15, SD = 0.33, t = 2.78, p = 0.009) and in flight (M = 0.36, SD = 0.40, t = 3.23, p = 0.007) (Fig 2).

### Does quantity of vehicles, type of vehicle and species identity influence time spent foraging, vigilant, or fleeing?

We found that ungulates increased the proportion of time spent foraging with the passage of a larger number of vehicles (compared to the passage of fewer vehicles; p = 0.015, Table 1A) and that they decreased the proportion of time fleeing (p = 0.011; Table 1C). Regarding vehicle size, we found that the passage of large vehicles (as opposed to passenger vehicles) results in decreased vigilance (p = 0.027, Table 1B) and increased flight (p = 0.013, Table 1C). There was also a non-significant trend that, with the passage of larger vehicles, ungulates reduced the proportion of time foraging (p = 0.131, Table 1A). Finally, we found that species identity plays a role in that, after vehicle passage, elk (compared to deer) spent less time fleeing (p = 0.027, Table 1C) and increased time allocated to vigilance (p = 0.013, Table 1B).

**Table 1:** Model results from deer and elk behavioral response to passing vehicles. Results from linear models examining if passing vehicles induced a change in the proportion of time deer and elk allocated to: A) foraging; B) vigilance (standing and looking); and C) flight at wildlife underpass on the Trans-Canada Highway, Banff National Park, Alberta.

A	Foraging behavior		
*Predictors*	*Estimates*	*CI*	*p*
Number of vehicles	0.18	0.04 – 0.32	**0.015**
Type of vehicle (Truck)	-0.24	-0.56 – 0.08	0.131
Species (Elk)	-0.26	-0.56 – 0.05	0.095
R^2^	0.396		
** B**	**Vigilance behavior**		
*Predictors*	*Estimates*	*CI*	*p*
Number of vehicles	-0.04	-0.14 – 0.07	0.468
Type of vehicle (Truck)	-0.26	-0.49 – -0.03	**0.027**
Species (Elk)	0.30	0.08 – 0.53	**0.009**
R^2^	0.354		
** C**	**Flight behavior**		
*Predictors*	*Estimates*	*CI*	*p*
Number of vehicles	-0.28	-0.49 – -0.08	**0.011**
Type of vehicle (Truck)	0.62	0.17 – 1.07	**0.013**
Species (Elk)	-0.59	-1.04 – -0.14	**0.016**
R^2^	0.655		

### Are changes in behavior (i.e., foraging, vigilance, flight) after vehicle passage correlated with the use, or disuse, of wildlife crossing structures?

When ungulates increased foraging (after vehicle passage) they were also more likely to use the nearby wildlife underpass (p = 0.038, Table 2A). Conversely, there were marginally-/non-significant trends where an increase in vigilance (p = 0.114, Table 2B) and flight (p = 0.09, Table 2C) behaviors were correlated with a reduced probability of underpass use.

**Table 2:** Model results from logistic regression of mitigation structure use and behavioral response to vehicles. Logistic regression results—probability of elk and deer crossing a road, using a wildlife underpass, as a function of species identity and the change in the proportion of time allocated to A) foraging; B) vigilance (standing and looking); and C) flight before vehicle passage compared to the proportion of time allocated after vehicle passage.

A		Use of crossing structure (foraging)		
*Predictors*	*Estimate*	*Odds Ratios*	*Odds Ratio CI*	*p*
Foraging	4.97	143.63	2.82 – 63126.92	**0.038**
Species (Elk)	1.97	7.14	0.58 – 225.22	0.169
R^2^ Tjur		0.385		
** B**		**Use of crossing structure (vigilance)**		
*Predictors*	*Estimate*	*Odds Ratios*	*Odds Ratio CI*	*p*
Vigilance	-2.01	0.13	0.01 – 1.39	0.114
Species (Elk)	0.10	1.10	0.22 – 6.19	0.907
R^2^ Tjur		0.108		
** C**		**Use of crossing structure (flight)**		
*Predictors*	*Estimate*	*Odds Ratios*	*Odds Ratio CI*	*p*
Flight	-5.04	0.01	0.00 – 0.58	0.090
Species (Elk)	-3.74	0.02	0.00 – 0.97	0.113
R^2^ Tjur		0.423		

## Discussion

Admittedly, our sample sizes were small, but this was to ensure unconfounded analysis. These results should therefore be considered somewhat preliminary. Overall, both white-tailed deer and elk responded to vehicle passage with a decrease in time allocated to foraging and an increase in both vigilance and flight behavior (Fig 2). Interestingly, elk increased vigilance while deer increased flight behaviors after vehicle passage (Table 1). A study that directly compared elk to mule deer (*O*. *hemionus*) found that deer were more likely to avoid roads than elk [23]. This may suggest that deer have less experience with traffic and thus could be less likely to habituate to traffic. Larger-bodied species, while initially more likely to be disturbed by humans, may also, be more likely to habituate to humans and human-related stimuli [24]. Thus, body size may explain the differences in the behavioral responses we found between deer and elk.

While overall, we found an increase in flight behavior after vehicle passage, we also found that time allocated to flight behavior decreased (while foraging increased) as more vehicles passed. This might suggest that animals living in areas where vehicle passage is more intermittent than constant are more vulnerable to disturbance by traffic. A previous study [10] found a similar negative relationship between elk flightiness and traffic volume. The mechanism underlying this could reflect the nature of the stimulus or reflect a habituation-like process. When more vehicles pass, they produce a louder sound that is detectable at a greater distance. Thus, animals might not be startled by an oncoming vehicle and have time to respond to it. Alternatively, animals may habituate to more vehicles, and this may be responsible for the attenuated responses. Prior work has shown that ungulates habituate to acoustic stimuli [25–30] and elk have been previously reported to habituate to people along roads and areas with other human activities [31, 32]. That said, we find that larger vehicles (i.e., semi-trucks) increase flight response; this may be because the substantively large stimuli associated with large vehicles, in line with prior research finding negative effects of motorized vehicles [33], simply overwhelms habituation.

**Fig 3:**
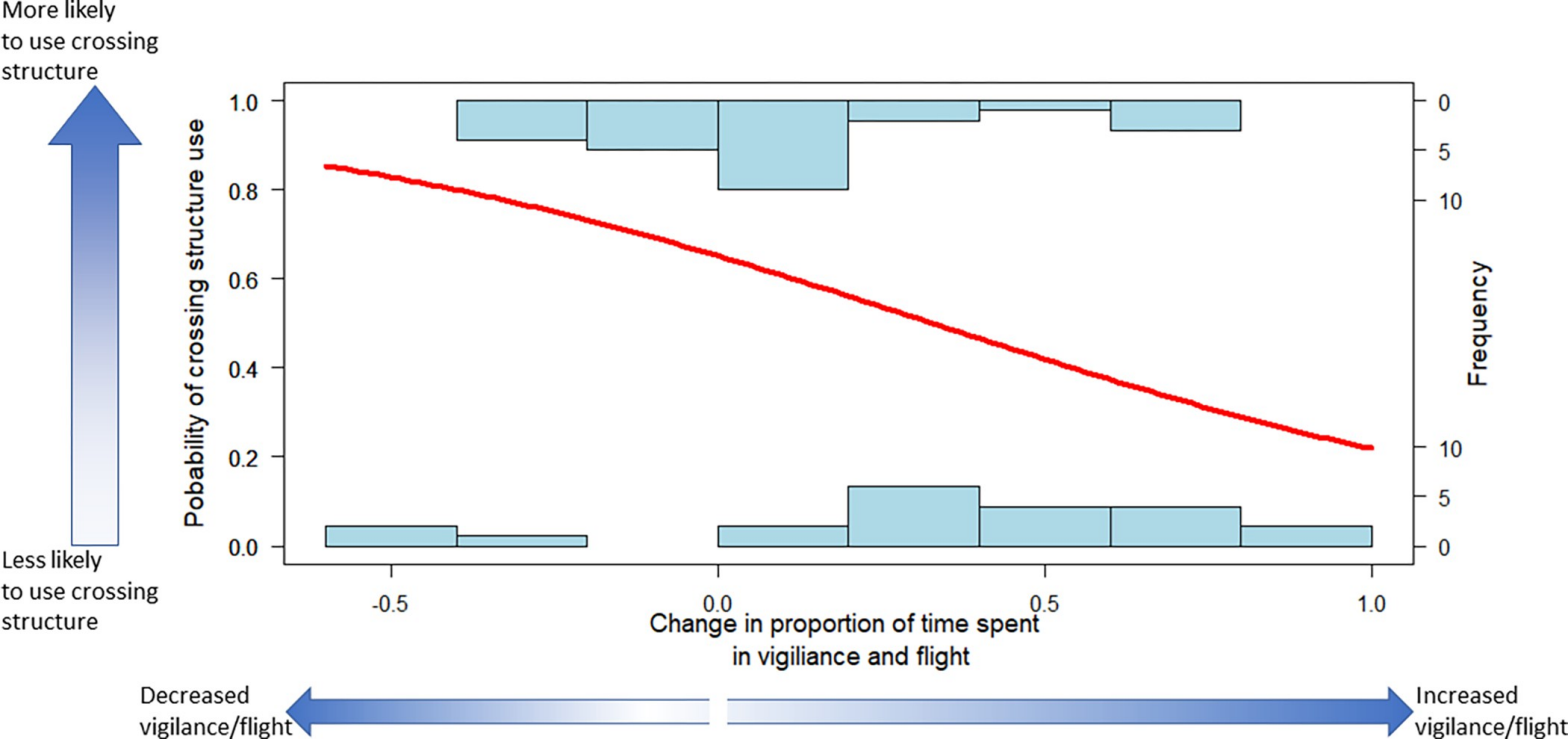
Results from logistic regression depicting variation in the probability of elk and deer crossing a road as a function of time allocated to vigilance & flight responses. Values, on the X-axis larger than zero indicate an increase in vigilance and flight behavior after vehicle passage (i.e., proportion of time spent on vigilant/flight behaviors after a vehicle passes minus the proportion of time spent on vigilant/flight behaviors before a vehicle passes). Larger values on the “probability of crossing” Y-axis, and the corresponding line in the figure, indicate increased probability of using a crossing structure. Deer and elk individuals are less likely to use a crossing structure after demonstrating vigilant/flight behavior in response to a vehicle. The “frequency of crossing” Y-axis shows the distribution of vigilance/flight in animals that either used the wildlife crossing structure (top) or those that did not use the crossing structure (bottom).

The observation that ungulates were more likely to use the underpasses when they were not disturbed by traffic suggests that traffic can affect connectivity at wildlife crossing structures (Fig 3, Table 2). Relatedly, we find a marginally significant trend where ungulates were less likely to use crossing structure after displaying flight (and vigilance) behaviors. Effects of traffic that influence ungulate behavior may have downstream effects of limiting or filtering the movement of individuals and potentially fragment populations as suggested by a previous study [10]. Importantly, however, animals might not cross during times of high-traffic, but could still cross when traffic decreases with fewer disruptions [10]. Thus, while immediate behavioral responses to traffic might affect the use of wildlife passage, traffic per se, may not [34]. Great potential exists for future studies to examine the association between behavioral responses and wildlife crossing rates at underpasses over longer periods of time to better understand how individuals and species are affected by vehicular traffic (e.g., by way of acoustic, visual, or olfactory stimuli), the duration of these effects and adaptation.

## Conclusions

Our finding of increased vigilance and flight in response to passing vehicles comes with a concomitant reduction in foraging (Fig 2). We also find that individuals who respond to passing vehicles with decreased vigilance/flight behaviors use crossing structures more frequently than their more vigilant counterparts. Time spent on vigilance, instead of foraging, and avoidance of crossing structures could negatively influence population connectivity and fitness in anthropogenic landscapes. Deleterious fitness effects could be especially pronounced when coupled with other aspects of human-modified landscapes. Conservation and management efforts should work to minimize the effect of roads, especially near corridors designed to permit wildlife movement, with the goal of having wildlife underpasses appear to wildlife as the safe haven they were intended to be [35]. This could be done as simple experiments using unaltered (control) wildlife underpasses with underpasses (treatment) that have sound walls or barriers that attenuate traffic noise levels, block lights and the view of oncoming traffic. Information from this type of study will contribute to our emerging knowledge of traffic impacts on crossing structure function and efficacy.

## Supporting information

S1 Data Spreadsheet used in analysis. Data included, in columns from left to right—unique identifier, difference in the proportion of time allocated to each of the following behaviors (calculated as time spent allocated to behavior X after vehicle passage/total time after vehicle passage)—(time allocated to behavior X before vehicle passage/total time before vehicle passage): “vigilance,” “forage,” “run”; species (either white-tailed deer or elk); number of vehicles; type of vehicle; binary value for if the animal utilized the passage corridor.
(CSV)
